# A Case of Perifollicular Macular Amyloidosis

**DOI:** 10.7759/cureus.29010

**Published:** 2022-09-10

**Authors:** Imran T Baig, Jamael L Thomas, Misha V Koshelev

**Affiliations:** 1 Dermatology, University of Texas Health Science Center at Houston McGovern Medical School, Houston, USA

**Keywords:** pruritus, macular amyloidosis, localized cutaneous amyloidosis, jak inhibitor, friction, eosinophils

## Abstract

Macular amyloidosis is a common type of primary localized cutaneous amyloidosis. We present a case report of a 74-year-old patient with no significant past medical history who was evaluated for dark macules and pruritus for over a year. On exam, follicular-based brown macules on the upper and lower back, bilateral shoulders, and bilateral dorsal upper arms were noted. The morphology and distribution of follicular-based macules was unusual, so the differential included follicular lichen planus, follicular eczema, and macular amyloidosis. Punch biopsy showed deposits of eosinophilic fibrillary material along with pigmentary incontinence in the papillary dermis, consistent with macular amyloidosis. Additionally, there was some trapping of the adnexal structures with atrophy of the periadnexal fat in the reticular dermis. In macular amyloidosis keratin, intermediate filaments such as cytokeratin serve as the amyloid precursors which deposit in the superficial dermis. Characteristically, macular amyloidosis presents as hyperpigmented macules or patches, often in a “rippled” linear pattern. This case highlights a rare presentation of macular amyloidosis because of the atypical follicular involvement and emphasizes the variety of presentations for localized cutaneous amyloidosis. Additionally, new treatment options such as Janus Kinase inhibitors and their potential role in the pathological pathway are discussed.

## Introduction

Macular amyloidosis falls under the broad category of primary cutaneous amyloidosis. In macular amyloidosis, amyloid is harmlessly deposited in the superficial dermis. The cause of macular amyloidosis is not completely understood, but it is associated with friction and scratching [1,2]. Although it is a relatively common condition, it can cause physical and psychological suffering secondary to pruritus. Macular amyloidosis is more commonly seen in people of Asian, Middle Eastern, and South American descent [3]. Clinically, it appears as hyperpigmented macules in a “rippled” linear pattern most commonly on the upper back and extensor upper extremities. Current treatment measures are aimed at controlling symptoms; however, recent advancements in Janus Kinase inhibitors may unlock novel therapies [4]. Expanding the clinical spectrum of this condition, we describe a unique case of macular amyloidosis with predominantly follicular involvement.

## Case presentation

**Figure 1:**
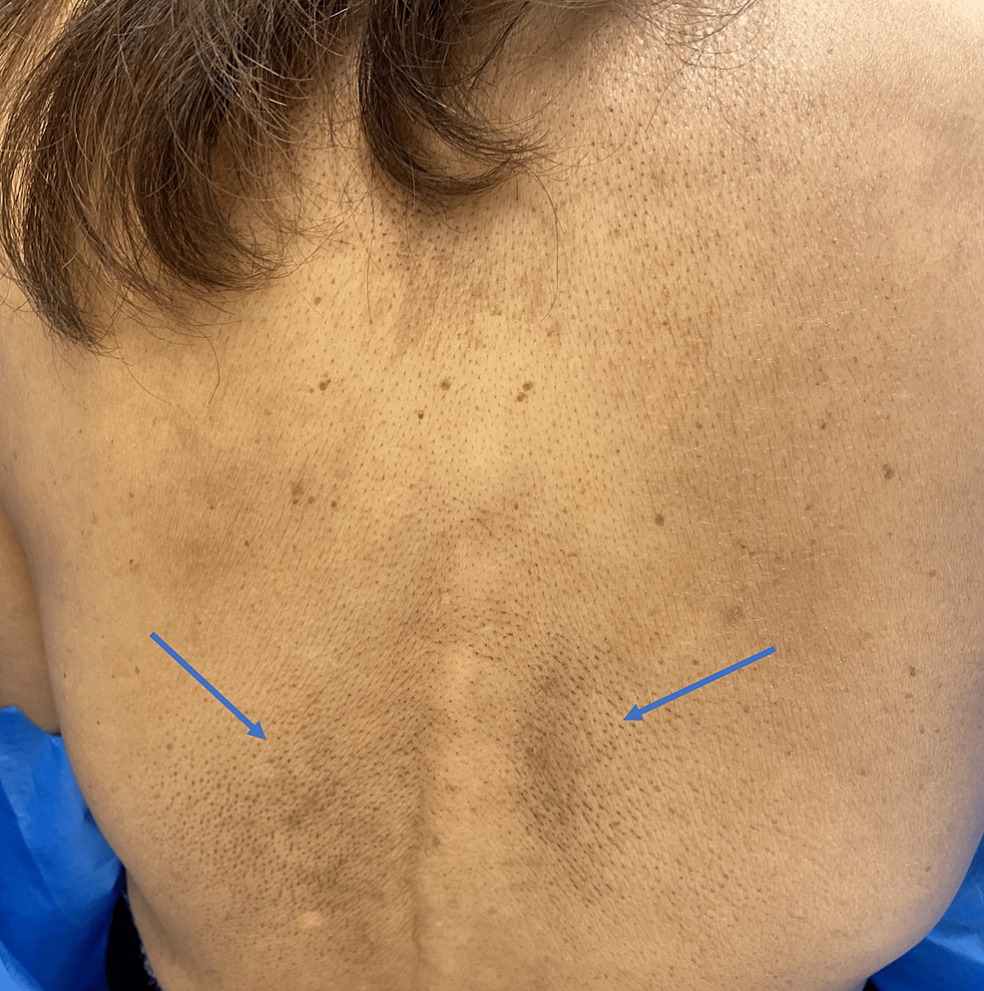
Perifollicular-based brown macules on the upper and lower back

**Figure 2:**
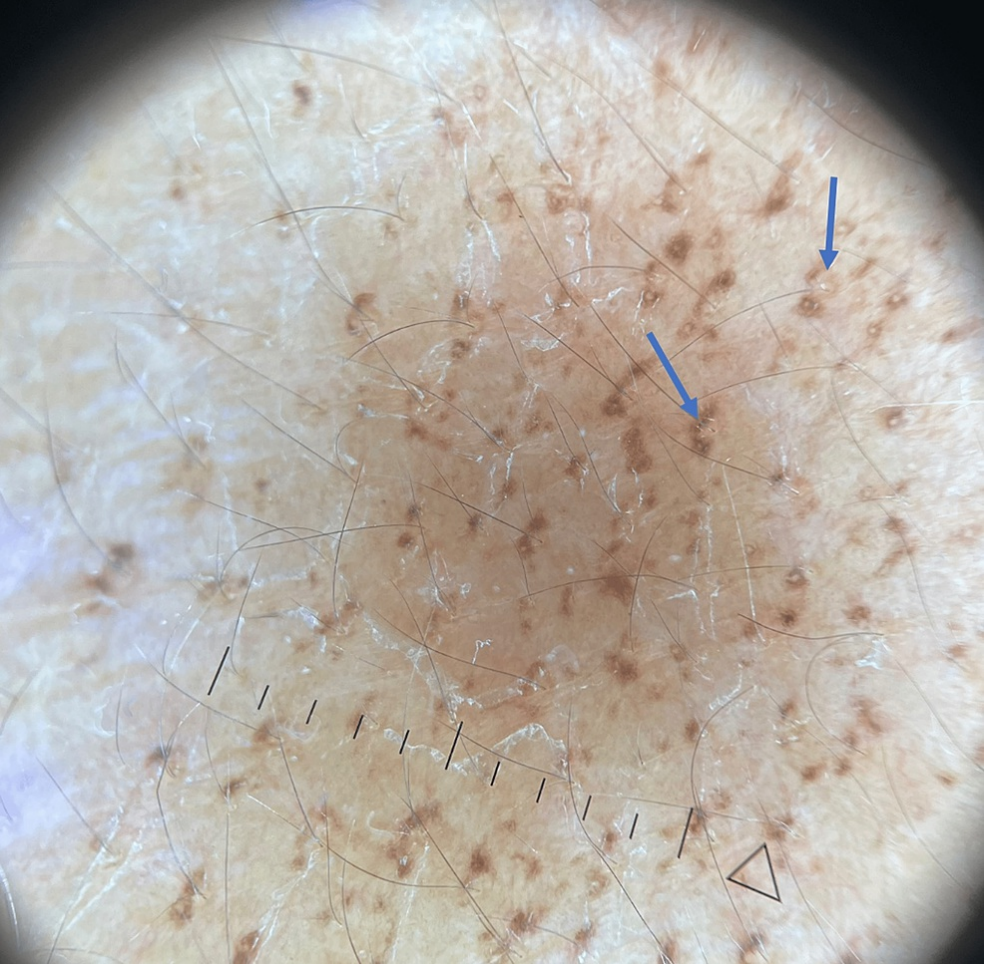
Dermoscopy of hyperpigmented macules showing dermal melanin focused around follicular ostia. Blue arrows demonstrate pigment with central clearing around follicle.

A 74-year-old Asian female patient with a past medical history of hypothyroidism on levothyroxine was evaluated by dermatology in February 2022 for dark marks and pruritus for over a year. The patient noted diffuse pruritus on her back which began several years before the rash appeared. The rash on her shoulder and arms was not particularly itchy. The patient denied redness prior to the dark spots appearing or a history of similar lesions in the past. The patient’s medications included levothyroxine for hypothyroidism and alendronate for osteoporosis but denied new medications. Physical examination revealed 1-2 mm follicular-based brown macules on the upper and lower back, bilateral shoulders, and bilateral dorsal upper arms (Figures 1, 2). The morphology and distribution of the follicular-based macules were peculiar, and diagnosis based on clinical presentation was difficult. The differential included follicular lichen planus, follicular eczema, and macular amyloidosis.

**Figure 3:**
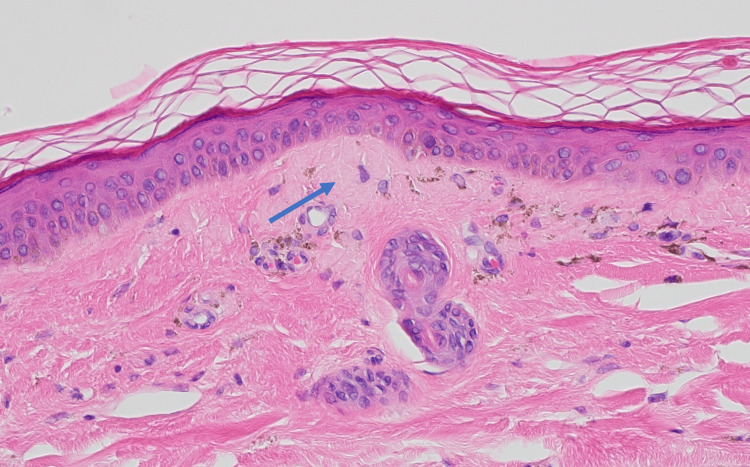
Histopathologic analysis showing sub-basilar amorphous eosinophilic deposits with melanin incontinence in the superficial dermis

**Figure 4:**
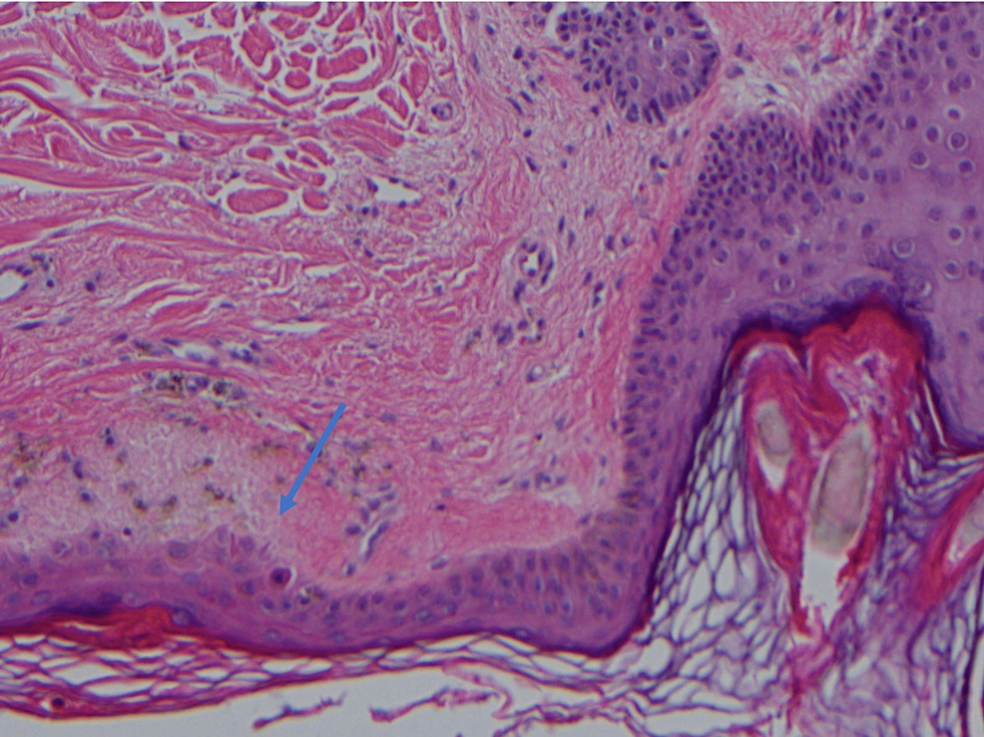
Histopathologic analysis showing a follicle with perifollicular amorphous eosinophilic deposit in papillary dermis accompanied by melanin pigment incontinence

A 4-mm punch biopsy of the right posterior shoulder was performed (Figures 3, 4). In the papillary dermis, there were deposits of eosinophilic fibrillary material along with pigmentary incontinence consistent with macular amyloidosis. In the reticular dermis, there was some trapping of the adnexal structures with atrophy of the periadnexal fat; however, deeper aggregates of mononuclear cells often seen in morphea and scleroderma were absent. Additionally, sections of the punch showed numerous round yeast forms in the stratum corneum, consistent with Malassezia yeast.

The patient was started on topical triamcinolone for the treatment of pruritus. The Malassezia was treated with two doses of oral fluconazole 300 mg seven days apart and ketoconazole shampoo to affected areas 2-3 days per week. The patient was scheduled to follow up in three months. At the three-month follow-up mark, the patient did not show up to her appointment but called to request a refill of triamcinolone cream for pruritus.

## Discussion

Localized cutaneous amyloidosis involves extracellular deposits of amyloid proteins in the skin without systemic involvement. The main types of localized cutaneous amyloidosis are macular amyloidosis, lichen amyloidosis, nodular amyloidosis, familial primary localized cutaneous amyloidosis, and secondary localized cutaneous amyloidosis. In macular amyloidosis, keratin intermediate filaments such as cytokeratin serve as the amyloid precursors which deposit in the superficial dermis. Although the etiology of macular amyloidosis is poorly understood, friction, itching, genetics, and environmental factors have been shown to have an association [1,2]. This disorder has a lower prevalence in western countries and is more commonly encountered in Asia and South and Central America [3]. Classically, macular amyloidosis presents as hyperpigmented macules or patches, sometimes in a “rippled” linear pattern. The most common areas for these lesions to appear are the upper back and extensor upper extremities. The lesions are usually pruritic but can also be asymptomatic.

This case presented a diagnostic dilemma because of the atypical follicular involvement (Table 1). The classic reticulated or rippled pattern was not noted. To our knowledge, there have not been any reported cases of macular amyloidosis presenting with follicular involvement. One differential diagnosis considered was follicular lichen planus due to the distribution of the macules. There has been one documented case of follicular lichen planus presenting on the trunk where a 34-year-old male presented with erythematous, keratotic, folliculocentric papules following Blaschko's lines [5]. However, it is rare for it to present in other areas of the skin without concurrent involvement of the scalp [6]. Follicular atopic dermatitis was also suspected due to pruritus and follicular distribution. It usually presents with a pattern of very pruritic follicular accentuation [7]. Given that the lesions in this case were flat, follicular eczema and follicular lichen planus were less likely. If the clinical presentation is still unclear, a biopsy can provide clinicopathological correlation to help confirm the diagnosis.

**Table 1:** Differential for patient with perifollicular macules

	Macular Amyloidosis	Follicular Lichen Planus	Follicular Atopic Dermatitis
Morphology	Hyperpigmented macules or patches (sometimes in a “rippled” linear pattern).	Scaly skin, redness around hair follicles	Cracked, dry or scaly skin
Symptoms	Itching	Itching, pain, burning on scalp	Itching, burning
Location	Upper back and extensor upper extremities	Scalp and hair	Chest, back, abdomen, and flanks
Causes	Combination of genetic and environmental causes with prolonged friction	Likely related to an inflammatory response mediated by T lymphocytes targeting follicular antigens	Genetics, epidermal barrier disruption, and dysregulation of the immune system
Treatment	Topical corticosteroids, topical calcineurin inhibitors	Topical, intralesional and oral corticosteroids	Lifestyle changes, moisturizers, topical corticosteroids

Since there is no potential for visceral involvement, management of macular amyloidosis is centered around treating its associated symptoms. Most importantly, patients should be counseled to avoid rubbing or scratching the affected areas. Potent topical corticosteroids can be applied one or two times a day to the affected areas. Patients who fail first-line therapy can try other methods such as calcineurin inhibitors, intralesional corticosteroids, systemic retinoids, and cyclosporine. Non-pharmacologic options include phototherapy, dermabrasion, and carbon dioxide laser [8]. Esmat et al. conducted a trial comparing superficial and deep methods of a fractional carbon dioxide laser and found that both reduced pigmentation, thickness, itching, and amyloid deposits, but the superficial mode was less painful [8]. Accurate recognition of macular amyloidosis is vital so that primary prevention of inciting factors, such as friction, and treatment targeted at the symptoms can be initiated immediately and provide relief to the patient.

Recently, developments in Janus Kinase (JAK) inhibitors are changing the landscape of dermatologic therapy [9]. Chen and Yang described a case of a 21-year-old Asian man with biopsy-proven primary cutaneous amyloidosis (PCA) that had failed multiple treatments directed at controlling his pruritus [10]. The patient was subsequently started on tofacitinib 5 mg twice a day and reported a dramatic improvement in pruritus and reduction in lesions in two weeks. Tofacitinib is an oral inhibitor that blocks JAK1 and JAK3 signaling and can be used for conditions such as atopic dermatitis and psoriasis [9]. Additional research suggests that JAK1 signaling in sensory neurons contributes to chronic itch [11]. Even though the mechanism of tofacitinib to relieve the symptoms of PCA is not completely understood, this case encourages further investigation of JAK inhibitors, and their potential uses in the setting of PCA. Furthermore, newer topical JAK inhibitors such as Ruxolitinib (JAK1/2), which is approved for atopic dermatitis, provide better safety profiles and should be the focus of future research regarding treatment modalities in conditions such as PCA [12].

## Conclusions

Macular amyloidosis is one of the more common types of primary cutaneous amyloidosis. It is usually diagnosed with its classic clinical presentation and confirmed with a biopsy displaying amyloid deposits within the skin. This patient’s presentation of follicular-based macules was atypical of macular amyloidosis. Therefore, a punch biopsy helped determine the underlying cause of the patient’s symptoms. Once the diagnosis of macular amyloidosis was confirmed, appropriate treatment for symptom relief was started. This unusual clinical picture emphasizes the variety of ways that macular amyloidosis may present.
